# An alternative catheter to invasive blood pressure monitoring in a patient with Takayasu arteritis undergoing open adrenalectomy: A case report

**DOI:** 10.1097/MD.0000000000045496

**Published:** 2025-10-24

**Authors:** Xiao Gu, Yunyan Wang, Zhibin Zhao, Ying Chen

**Affiliations:** a Department of Anesthesiology, Lianyungang Clinical College of Nanjing Medical University, Lianyungang City, China; b Department of Anesthesiology, The First People’s Hospital of Lianyungang, Lianyungang City, China.

**Keywords:** catheterization, invasive blood pressure monitoring, Takayasu arteritis

## Abstract

**Rationale:**

Continuous invasive blood pressure monitoring is crucial during the perioperative period, particularly in high-risk and critically ill patients. In patients with Takayasu arteritis (TAK) and severe vascular stenosis, conventional arterial cannulation is often technically challenging because of vessel occlusion or fragile tissue. This case report explores the feasibility of using a central venous catheter instead of an arterial puncture kit for invasive blood pressure monitoring when standard arterial catheterization is impractical.

**Patient concerns:**

A 56-year-old female with a history of TAK presented for adrenalectomy.

**Diagnoses:**

Medical imaging revealed an adrenal tumor and TAK with multi-territorial vascular involvement (radial, brachial, and dorsalis pedis arteries).

**Interventions:**

The patient underwent adrenal tumor resection. Continuous invasive blood pressure was monitored under ultrasound guidance using an 18-gauge central venous catheter for femoral artery puncture.

**Outcomes:**

The procedure was completed uneventfully with timely hemodynamic monitoring guided by reliable invasive measurements.

**Lessons:**

This alternative method has the potential to reduce the risk of intraoperative catheterization and ensure reliable intraoperative blood pressure monitoring when conventional puncture is impossible.

## 1. Introduction

Takayasu arteritis (TAK) is a rare, nonspecific vasculitis that involves the aorta and its major branches. This condition progressively affects the entire blood vessel layer of the vascular adventitia.^[1]^ As a result, the vessel wall thickens significantly and the vessel lumen may narrow or even become occluded. For patients requiring surgical intervention, the challenge of invasive monitoring of arterial blood pressure arises when there are several arterial stenoses in the upper and lower extremities. Few reports have elaborated on management considerations for the occurrence of such events. This report describes the successful management of arterial monitoring in patients with TAK through cannulation of the femoral artery using an alternative catheter.

## 2. Case presentation

**Figure 1.**
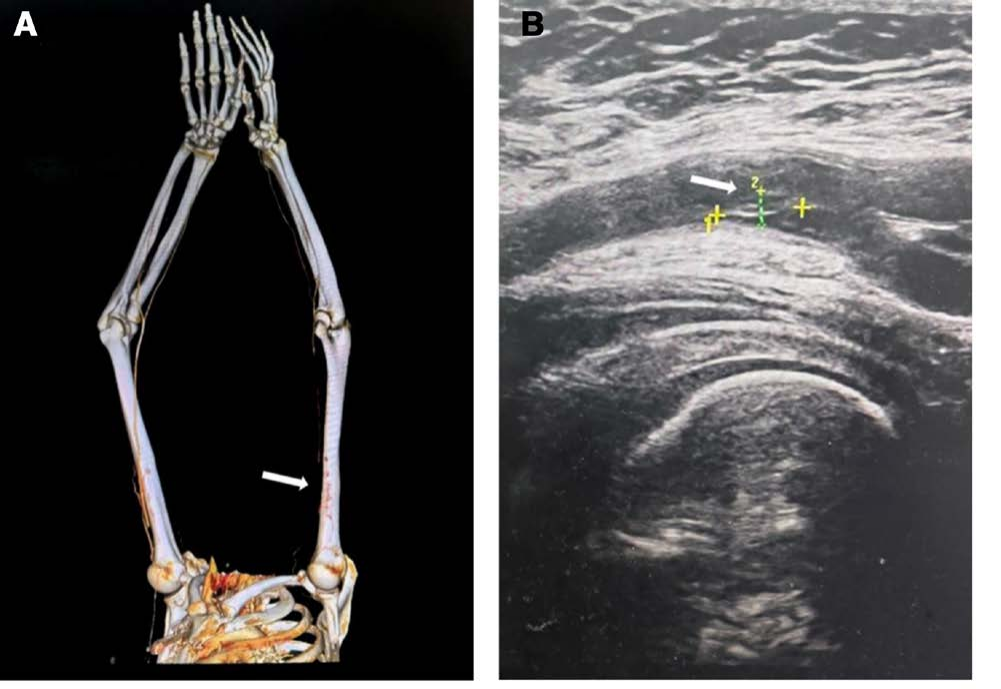
Results of medical imaging examination. (A) Vascular CTA of the upper extremity. There is a long-segment occlusion of the left axillary artery and the origin of the brachial artery (white arrow). (B) Vascular Doppler ultrasound of the lower limbs. Hypoechoic plaque is detected in the left femoral artery (white arrow). CTA = computed tomography angiography.

A 56-year-old woman underwent adrenal tumor resection surgery at our hospital. During an incidental examination 2 months ago, she was found to have a left adrenal mass and was highly suspected to have pheochromocytoma or paraganglioma. The patient had a history of TAK with multiple preexisting stenoses and occlusions. Preoperative upper-extremity computed tomography angiography (CTA) showed non-calcified plaques with severe stenosis in the bilateral subclavian arteries and the right axillary artery. There was a long-segment occlusion of the left axillary artery and the origin of the brachial artery (Fig. 1A). The distal end of the left brachial artery was slender and faint. The left deep palmar arch was slightly pale and sparse. Doppler ultrasound revealed a blunted flow spectrum and an increased resistance index in the bilateral renal arteries. The intimal surfaces of the carotid, femoral, and popliteal arteries were rough. A hypoechoic plaque was detected in the left femoral artery, measuring approximately 8.6 × 2.9 mm (Fig. 1B). No significant blood flow signal was observed bilaterally within the lower end of the superficial femoral artery or the upper end of the popliteal artery.

**Figure 2.**
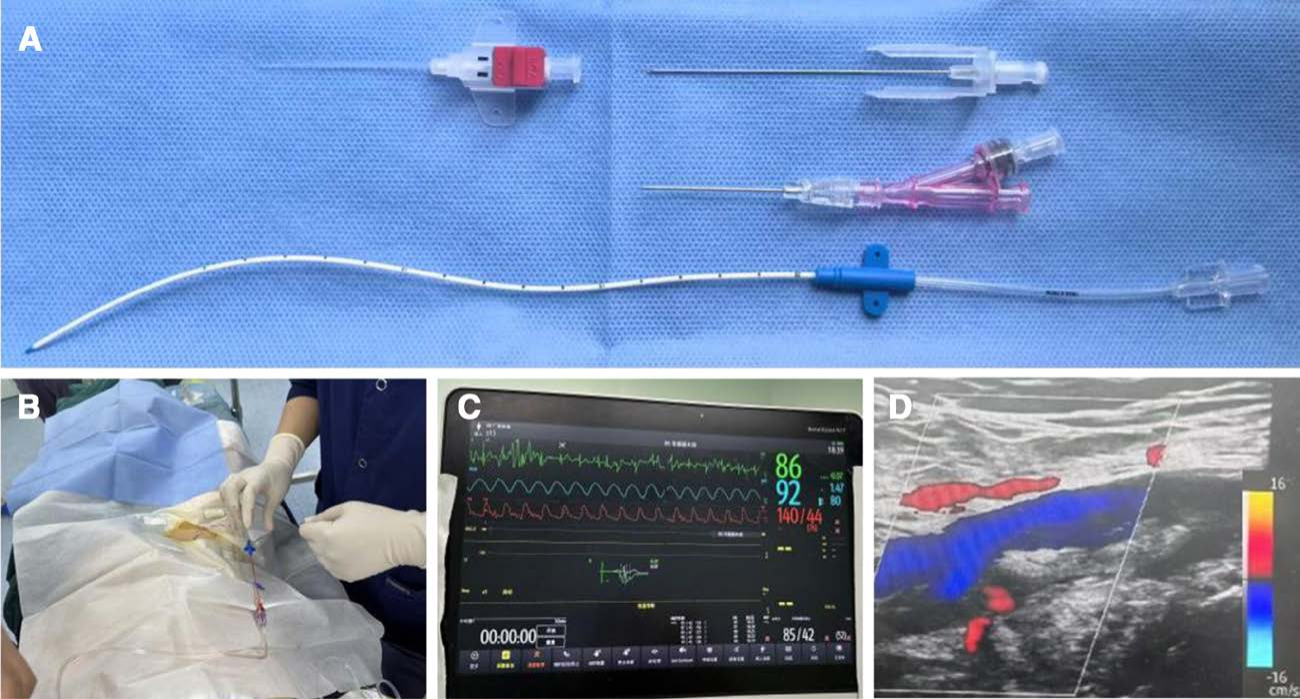
Femoral arterial access and blood pressure monitoring during surgery. (A) 20-gauge arterial cannula (upper) and 18-gauge central venous catheter (lower). (B) Cannulation of the right femoral artery with an 18-gauge single lumen intravenous catheter. (C) Arterial waveforms are displayed on the electrocardiographic monitor. (D) Doppler ultrasound of the femoral artery following catheter removal. The vessel was patent with normal flow, and no other complications were observed.

The patient was scheduled to undergo general anesthesia. When the patient entered the operating room, vital signs were routinely monitored, including heart rate, blood pressure (although noninvasive measurement was not possible due to TAK), pulse oxygen, and BIS. Subsequently, an open peripheral vein of the upper limb was accessed. Prior to puncture, we found that the pulses of the radial, brachial, and dorsalis pedis arteries were not palpable. Ultrasound evaluation of the patient’s blood vessels showed that the lumen of the arteries was narrow and tortuous. Given the increased risk of vasospasm, thrombosis, and even limb ischemic necrosis if we continued to puncture these arteries, we decided against using them for arterial puncture. Fortunately, the right femoral artery of the patient showed a favorable lumen on ultrasonography; therefore, we opted to perform invasive blood pressure monitoring via the femoral artery. However, the average depth of the femoral artery from the skin surface is 3 to 4 cm, whereas the length of the conventionally used 20-gauge arterial puncture needle is 4.5 cm. Under these circumstances, if catheterization is performed, the portion of the intra-arterial catheter entering the vessel would be too short. Furthermore, considering the changes in position (the patient was placed in the lateral position with the operating table placed in maximal flexion during surgery) and other influencing factors, the intra-arterial catheter was prone to bending or even becoming dislodged. This could compromise the accuracy of the results obtained from invasive blood pressure monitoring. Therefore, we chose to use a central venous catheter (CVC) as an alternative to an arterial puncture kit for invasive blood pressure monitoring. An 18-gauge single-lumen CVC, 20 cm in length, from the Central Venous Catheter Kit (SCW Medicath Ltd., Shenzhen, China) was used for the femoral lines (Fig. 2A and B).

The catheter was inserted using the Seldinger technique under ultrasonographic guidance. Next, the catheter was connected to an arterial transducer (DPT-248, SCW Medicath Ltd.), which was then linked to the lead wires of the electrocardiographic monitor (BeneVision N17, Mindray Bio-Medical Electronics Ltd., Shenzhen, China). The transducer was placed at the same level as that of the patient’s right atrium, and the zero-point reference was calibrated. Additionally, the adequacy of the damping was assessed using a fast-flush test. At the point of insertion, the catheter was secured with sutures and then covered with a sterile, transparent, and occlusive dressing.

**Figure 3.**
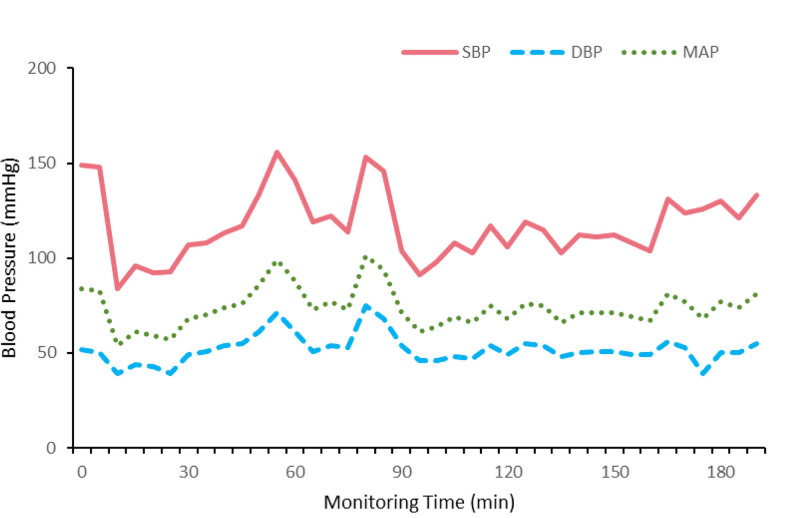
Blood pressure trend chart. The changes of invasive blood pressure throughout the monitoring period. DBP = diastolic blood pressure, MAP = mean arterial pressure, SBP = systolic blood pressure.

Invasive arterial blood pressure was measured after observing a normal waveform (Fig. 2C). No catheter dislodgment occurred during surgery. During surgery, invasive blood pressure readings obtained from the femoral arterial site remained steady (Fig. 3), with the mean systolic blood pressure (SBP) at 117 mm Hg and the mean arterial pressure at 73 mm Hg throughout the monitoring period. The blood pressure waveform was optimal and the patient maintained stable intraoperative hemodynamics. The perioperative course was uneventful. After removal of the venous catheter, a Doppler ultrasound was performed and showed no evidence of femoral artery injury (Fig. 2D). The patient was observed in a high-dependency unit for 1 hour and then shifted to the ward. Postoperative pathological diagnosis confirmed that the lesion was a paraganglioma.

## 3. Discussion

Arterial blood pressure is a fundamental cardiovascular parameter and inaccurate arterial pressure measurements can lead to inappropriate interventions by anesthesiologists.^[2,3]^ Invasive blood pressure is the gold standard for arterial blood pressure assessment in critically ill patients and in those undergoing major surgical procedures.^[4]^ Based on the patient’s vascular anatomy, the femoral artery was selected as the puncture site following a thorough risk-benefit analysis. Notably, we opted for an 18-gauge CVC instead of the conventional 20-gauge catheter.

It is widely recognized that optimal waveform quality is essential for accurate arterial blood pressure measurements. The pressure measurement system primarily consists of an arterial catheter, fluid-filled extension tube, pressure transducer, and continuous flushing device. Each pressure measurement system had a unique natural frequency and damping coefficient. When the arterial waveform exhibits improper dynamic responses, such as underdamping and overdamping, this leads to discrepancies between the actual and displayed blood pressure values. Underdamping can result in overestimation of SBP and pulse pressure (PP), along with underestimation of diastolic blood pressure. On the other hand, overdamping may lead to an underestimation of SBP and PP and an overestimation of diastolic blood pressure.^[5–7]^

A previous study showed that the length and inner diameter of the catheter influence the damping properties of a BP measurement system.^[8]^ Twenty-gauge catheters have been shown to be less affected by underdamping than 18-gauge CVC. In contrast, another study reported no statistically significant differences in the final SBP/PP measurements across catheter sizes, despite observable variations in the system frequency response.^[9]^ In this study, we used an 18-gauge CVC for invasive blood pressure monitoring. No abnormal changes were observed in the arterial waveforms. It can be inferred that the system performs effectively, and that the blood pressure measurement errors are within acceptable limits. Therefore, the 18-gauge CVC can be used as a temporary alternative to the conventional arterial catheter in patients requiring femoral artery measurements when no other suitable method is available.

This study has several limitations. First, the catheters exhibited variability in both length and diameter, which may confound the interpretation of the dynamic response differences. Although the statistical significance of these variations requires further investigation, it remains unclear whether such differences could directly account for discrepancies in blood pressure measurements. Second, the material disparity between the 18-gauge CVC and the 20-gauge arterial catheter used in this investigation may have affected the dynamic response characteristics of the pressure measurement system. Consequently, the clinical acceptability of the observed pressure errors necessitates rigorous validation using supplementary experimental protocols.

This case provides a critical lesson on ensuring reliable hemodynamic monitoring in extreme clinical scenarios. In high-risk situations where noninvasive monitoring is unavailable and vascular access is challenging, such as in the patient with TKA, the femoral artery can be cannulated with an 18-gauge CVC to obtain a high-fidelity arterial waveform. The catheter must be securely anchored immediately after insertion to prevent early dislodgement. Furthermore, the surgical team must be explicitly informed of its critical role to avoid compromising this sole source of blood pressure monitoring. Additionally, the 18-gauge CVC offers advantages of soft texture and sufficient length, which minimizes vascular irritation and effectively prevents catheter dislodgement. We therefore hypothesize that this approach could be extended to specific critically ill patient populations, including those in whom arterial catheter placement has failed or those at risk of accidental dislodgement due to intraoperative position changes.

## 4. Conclusion

In patients with TAK requiring femoral arterial puncture for arterial pressure measurement, the use of an 18-gauge CVC as an alternative when an appropriately sized arterial catheter is unavailable may represent a viable clinical approach, although careful consideration of the potential hemodynamic effects is warranted.

## Author contributions

**Conceptualization:** Ying Chen.

**Data curation:** Xiao Gu.

**Investigation:** Xiao Gu, Yunyan Wang.

**Supervision:** Zhibin Zhao.

**Writing – original draft:** Xiao Gu, Yunyan Wang.

**Writing – review & editing:** Ying Chen.
